# Bardet-Biedl syndrome: The longer we miss, the worse is the outcome

**DOI:** 10.22088/cjim.13.4.805

**Published:** 2022

**Authors:** Michael Edwar, Usama Ragab, Ahmed Atia Kamel

**Affiliations:** 1 Department of Internal Medicine, Faculty of Medicine, Zagazig University, Zagazig, Egypt

**Keywords:** Bardet-Biedl syndrome, Ciliopathies, Consanguinity, Polydactyly

## Abstract

**Background:**

Bardet-Biedl syndrome (BBS) is characterized by obesity, cognitive abnormalities, rod-cone dystrophy, skeletal abnormalities, and many other secondary features.

**Case Presentation:**

We describe a 28-year-old man presented with postaxial polydactyly, retinitis pigmentosa, obesity, hypogonadism and learning difficulties. Renal insufficiency in form of acute kidney injury was the presenting feature and this explain the worse outcome. The diagnosis was delayed despite being classic. This delay in diagnosis leads to a lot of complications that worsen the patient's condition.

**Conclusion:**

The characteristics of BBS should be noted by doctors because an early diagnosis will result in a better outcome. The case was prone to numerous consequences due to the delay in diagnosis, which could have been avoided if an early diagnosis had been established.

Bardet-Biedl syndrome (BBS) is an autosomal recessive syndrome first described by two separate physicians, *George Bardet* formerly described a case with a cognitive abnormality, obesity, retinal dystrophy, renal dysfunction, hypogonadism, and postaxial polydactyly in 1920 (1). *Aruther Biedl*, an Austrian physician found the same criteria in different individuals two years later. The syndrome was coined later as Bardet – Biedl syndrome **(**2**)**. BBS is sometimes confused with another syndrome called Laurance – Moon syndrome, which was mentioned in 1866. Nowadays, it is widely accepted to be two different syndromes with some phenotypic resemblance. Both share obesity, retinal dystrophy, hypogonadism, renal affection, however, Laurance – Moon syndrome is distinguished with spastic paraplegia and BBS is characterized by postaxial polydactyly **(**2**, **3**)**. The prevalence of BBS is estimated in different populations and showed marked variation, wherein the European community ranged from 1: 125000 to 1: 160000. In the Middle East, also the prevalence varied wherein Bedouin Kuwait was estimated to be 1: 13000, however, in Tunisia, the prevalence was 1:156000 (4). 

This variation could be attributed to the discrepancy in consanguine marriage (5). BBS is a ciliopathy with a heterogeneous genetic base, up till now 16 genes have been identified. Mutation in BBS gene 1-14 is responsible for about 75 % of the affected individuals (6). In Egypt, no definitive data is available regarding BBS prevalence. This attributed to the lack of definitive screening tools and subsequently underestimation of this syndrome. So, the number of cases reported in Egypt may underestimate the real problem. 

Moreover, physicians being unaware of BBS syndrome, the diagnosis of such cases mostly is delayed till complications - especially renal and cardiovascular - present and this delay puts the patients with BBS at high risk of morbidity and mortality. In our report, we described the clinical, endocrinological and imaging evaluation of a 28-year-old man with a typical BBS that was not diagnosed till the age of 28.

## Case presentation

A 28-year-old Egyptian man born of consanguineous marriage presented complaining of pedal edema of two months duration that was associated with scrotal swelling and abdominal distension. During his childhood, his mother noticed that he had delayed motor and mental milestones together with childish behavior and delay in speech. He did not attend school due to learning difficulties and was considered by his parents as being mentally retarded. He started to develop visual acuity drop that was marked by stumbling while walking in the home since he was 12 years old, now on the assessment of his visual acuity, the patient can only count fingers and the visual field is restricted to tunnel vision. He has delayed sexual characters. He was diagnosed with type 2 diabetes mellitus two years ago, for which he started a premixed insulin regimen. He started to develop shortness of breath with lower limb edema, of gradual onset, slow progressive course, of pitting nature and not tender. The swelling extends above the knee, associated with scrotal swelling and abdominal distension. A week ago, the patient started to complain of repeated vomiting of gradual onset, progressive course, not related to meals, of no diurnal variation, associated with nausea and hiccough, and slightly improved with antiemetic drugs. The patient sought medical advice and investigated with routine laboratory investigations that revealed rising serum creatinine and urea, so the patient referred to Zagazig university hospitals to accomplish his investigations and start the treatment accordingly.

**Figure 1:**
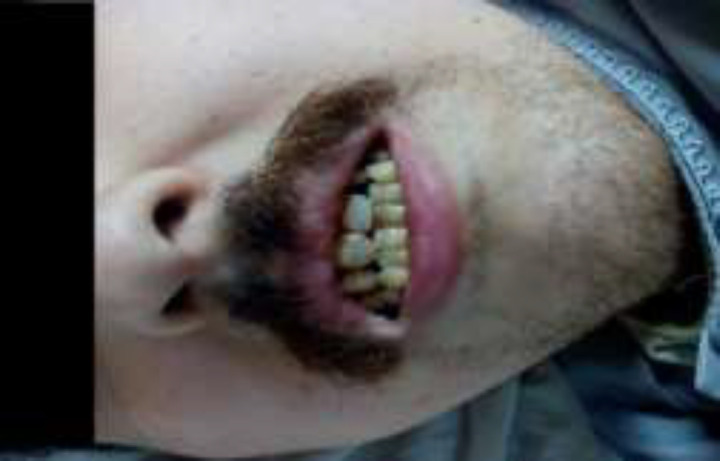
Facial features of patient with BBS

**Figure 2:**
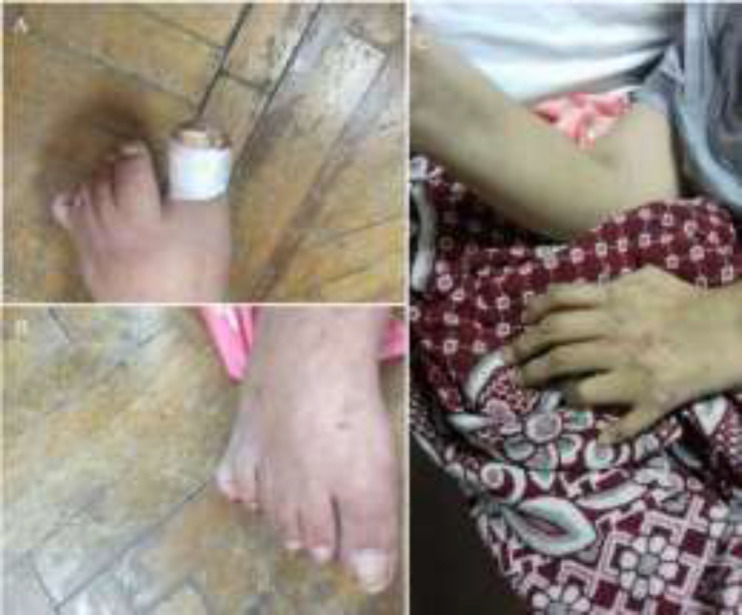
Skeletal features of BBS, A: polydactyly in left foot; B: syndactyly in right foot; C: accessory finger in left hand

He was 189 cm tall and weighed 112 kg, with a body mass index of 31.5 kg/m^2^. He had marked central obesity, a triangular elongated face, with frontal baldness, silky hair*,* crowded teeth and high arched palate (figure 1), his acral parts showed accessory fingers and toes accordingly (polydactyly). Fifth and sixth finger in each hand showed clinodactyly. Fifth and sixth toes in each foot were fused (syndactyly) (figure 2). The skin of the four limbs was rough and showing scales on scratching. Lower limbs were showing bilateral pitting painless edema, extending above the knee. Nails showed trophic changes. As for his back and genitalia, there was painless scrotal swelling observed. Both testes were present but were of small size when compared with testes of the same age by orchidometer (of almond size). 

The penis was of micro-size (stretched penis length is 2.4 cm) and pubic hair was sparse. Axial hair was scanty. Cardiovascular examination showed apex in the fifth left intercostal space mid-clavicular line, audible first and second heart sounds (was faint), no murmurs were noticed. As regards neck veins; they were not congested. Abdominal examination shows no organomegaly. Neurological examination of the patient showed low intelligent quotient (IQ) (subnormal mentality), and childish behavior, on the assessment of visual acuity, patient could only count fingers, the visual field was restricted to tunnel vision, rest of the neurological examination was normal.

Dilated fundus examination showed retinitis pigmentosa and bilateral optic atrophy. Routine investigations revealed microcytic hypochromic anemia, high blood glucose, elevated serum creatinine (6.98 mg/dl), high erythrocyte sedimentation rate and c-reactive protein, normal bilirubin, and low serum albumin (2.89 g/dL). Thyroid-stimulating hormone (TSH) was within normal range with high follicle-stimulating hormone (FSH) and luteinizing hormone (LH). Urine analysis showed proteinuria that was quantified by urinary protein to creatinine ratio (1850 mg/gm). Arterial blood gases revealed uncompensated metabolic acidosis.

Pelviabdominal ultrasound examination showed that liver was average size and of normal texture, portal vein patent of average caliber, spleen diameter was 14.6 cm, both kidneys are in place, enlarged, fair corticomedullary differentiation, right kidney showed mild backpressure, left kidney showed moderate back pressure, however, no stones could be detected. High-resolution spiral computed tomography (CT) was done, the presence of backpressure was confirmed, and no stones could be detected.

Echocardiography showed normal internal chamber dimensions, good systolic function, and mild pericardial effusion. The plan of management was focused on dealing with acute kidney injury, following-up kidney functions, searching for the other possible complications of BBS to deal with. The patient received three sessions of hemodialysis to correct uremia and acidosis with a blood transfusion of two units of packed RBCs to correct anemia, however during the follow-up of his kidney function, serum creatinine rose again and blood gases revealed acidosis, so the patient was prepared for permanent vascular access for regular hemodialysis. 

## Discussion

BBS is a rare ciliopathy of autosomal recessive inheritance that mostly occurs in children born from consanguineous marriages, it is characterized by obesity, post-axial polydactyly, learning difficulties, retinal dystrophy, and hypogonadism (7). Features are sometimes categorized into primary (that was previously mentioned) and secondary features that include speech delay, language disorders, strabismus, cataract, other skeletal anomalies such as brachydactyly or syndactyly, delayed mental milestones, diabetes mellitus, diabetes insipidus, hepatic fibrosis and structural heart disease (8). Thyroid dysfunction, congenital megacolon, epilepsy, anal stenosis, and abnormal dentition are rare associations. The diagnosis could be as early as antenatal period if suspected on antenatal imaging, if not; it will not be diagnosed till the patients start to develop frank phenotypic features most likely visual symptoms such as retinitis pigmentosa. *Beales et al*. revised diagnostic criteria indicate that a clinical diagnosis includes either four primary features or three primary and two secondary features. The primary and secondary clinical characteristics and their frequency in BBS are simplified in table 1 (8-10).

Classically, renal affection in BBS has been associated with polycystic kidney disease, a typical feature of ciliopathies. The prevalence of renal affection in BBS has been estimated at 53–82% (11). A recent study has estimated that half of the patients with BBS will develop functional renal disease and demonstrate that cystic or dysplastic disease only accounts for 30% of patients with renal disease, where the remainders have hydronephrosis, atrophic kidneys, or loss of corticomedullary differentiation. Around 8% of patients go on to develop end-stage renal disease requiring dialysis or transplantation. The presentation of renal disease in some patients develop as acute kidney injury in adulthood for unknown reasons, and a further group of patients develops end-stage kidney disease as a result of comorbidities including type 2 diabetes and hypertension (12). 

**Table 1:** Criteria for BBS diagnosis modified by Beales *et al*

**Primary Features**	**Frequency %**	**Secondary Features**	**Frequency %**
Rod-cone dystrophy	93	Developmental delay	50–91
Obesity	72–92	Dental anomalies	51
Genital anomalies	59–98	Diabetes mellitus	6–48
Polydactyly	63–81	Speech delay	54 – 81
Learning difficulties	61	Brachydactyly/syndactyly	46–100/8– 95
Renal anomalies	53	Anosmia/hyposmia	61
		Ataxia/poor coordination	40 – 86

Four primary features or three primary features plus two secondary features are required for a clinical diagnosis of Bardet–Biedl syndrome (8, 9, 10). Our patient had postaxial polydactyly, retinitis pigmentosa, obesity, hypogonadism, learning difficulties, renal insufficiency, mental delay, speech delay, dental anomalies, syndactyly and type 2 diabetes mellitus i.e., six primary and five secondary clinical features, which make our case a typical example of BBS. Our patient was presented to us with acute kidney injury, however without a prior diagnosis of BBS. Many renal abnormalities could lead to renal dysfunction with the development of chronic kidney disease with frequent acute on top exacerbation. In this patient, laboratory findings revealed non-nephrotic range proteinuria with urinary albumin to creatinine ratio of (1850 mg/gm), which may be attributable to diabetic kidney disease, however in context of absence of typical findings on fundus examination, this may be due to chronic kidney disease that may be associated with urinary protein loss. Guidelines for the treatment of BBS recommend that any patient should get a baseline renal ultrasound examination to determine the possibility of any abnormalities, however some patients with pathological renal ultrasound may not develop chronic kidney disease. (13) In our case however, we did not find any pathologic abnormality based on ultrasonographic findings apart from poor corticomedullary differentiation, and bilateral backpressure that may denote obstructive pathology. It is estimated that 6% of the adult BBS population reported urological problems requiring specialized management. Urological problems involved neuropathic bladder, vesico-ureteric reflux, urinary incontinence, and bladder outflow obstructions. (12)

Despite being a classic case of BBS, that fulfills most of the diagnostic criteria, the diagnosis was established by the age of 28 years. This delay in diagnosis made the case vulnerable to many complications that could be reversible if an early diagnosis was done. It should be of noted that there may be an overlap between phenotypic features of BBS including retinal involvement and skeletal anomalies and certain other genetic disorders such as Alström syndrome (distinguished from BBS by preserved cognitive function and absence of polydactyly), Meckel syndrome (distinguished from BBS by occipital encephalocele and other central nervous system anomalies) and McKusick-Kaufman syndrome (distinguished from BBS with the absence of retinal disease, obesity, and developmental disabilities). (14)

We guided patient relatives to undergo genetic testing; however, cost issue was a problem in this regard. On a one- month follow-up of regular hemodialysis, the pedal edema improved, and vomiting disappeared. The patient was given an identification card as BBS syndrome, his mother was counseled about BBS nature and about the necessity of follow-up to delay the disease complications as much as possible. As regards fertility issue in BBS patients, a recent article, by *Koscinski et al.*, has stated that despite severe obesity which is a primary feature in BBS, the hypothalamic-pituitary-gonadal axis will function properly in adults, sperm has normal motility, moreover, its centriole will perform the first division of zygotes, this represents a hope for future fecundability in BBS patients. (15)

**Learning objectives**

- Physicians should give attention to features of BBS, as early diagnosis will improve the outcome.
- Screening for retinitis pigmentosa by fundus examination should be carried on patients with unexplained skeletal deformities such as polydactyly or syndactyly to establish the diagnosis.
- Cardiovascular and renal complications responsible for most of morbidities and mortalities in BBS, so echocardiography, renal function tests, and renal imaging should be the screening and the follow-up tools in the diagnosed patients.
- Diabetes, hypertension, and other metabolic co-morbidities should be controlled intensively to reduce damage to other organ systems often implicated in BBS, such as the kidney and retina
- Consanguineous marriage is a strong risk factor for many inherited diseases including BBS, so genetic counseling is recommended in the case of consanguinity.
